# Detection of Exosomes Using Total Internal Reflected Imaging Ellipsometry

**DOI:** 10.3390/bios11050164

**Published:** 2021-05-20

**Authors:** Haoyu Liu, Wei Liu, Gang Jin

**Affiliations:** 1 NML, Institute of Mechanics, Chinese Academy of Sciences, 15 Bei-Si-Huan West Road, Beijing 100190, China; liuhaoyu@imech.ac.cn (H.L.); liuwei@imech.ac.cn (W.L.); 2 School of Engineering Sciences, University of Chinese Academy of Sciences, 19 Yu-Quan Road, Beijing 100049, China

**Keywords:** TIRIE, exosomes, surface protein, CD9, CD63, interaction kinetics

## Abstract

Exosomes are a kind of membrane-bound phospholipid nanovesicle that are secreted extensively in a variety of biological fluids. Accumulating evidence has indicated that exosomes not only communicate with cells, but also perform functional roles in physiology and pathology. In addition, exosomes have also elicited a great deal of excitement due to their potential as disease biomarkers. Therefore, requirements for sensitive methods capable of precisely and specifically determining exosomes were needed. Herein, we not only develop a sensing surface to capture exosomes but also compare two surface proteins on exosomes, which are appropriate for detecting exosome surface markers by total internal reflected imaging ellipsometry (TIRIE). Protein G and antibody were immobilized on a thin layer of golden substrate to form the biosensing surface. The bio-interaction between antibodies and exosomes was recorded by the TIRIE in real time. The distance between exosomes adhered on a surface was 44 nm ± 0.5 nm. The $K_{D}\;$ of anti-CD9 and exosome was lower than anti-CD63 and exosome by introducing pseudo-first-order interaction kinetics, which suggested that CD9 is more suitable for exosome surface markers than CD63. The limit of detection (LOD) of TIRIE was 0.4 μg/mL. In conclusion, we have proposed a surface for the detection of exosomes based on TIRIE, which can make the detection of exosomes convenient and efficient.

## 1. Introduction

Exosomes are membrane-bound phospholipid nanovesicles (30–100 nm in diameter) extensively secreted by cells of human body fluid, including saliva, urine, and blood [1]. Over the past few years, more and more evidence has suggested that exosomes are like signaling carriers, which have proteins, lipids, and genetic material [2]. Additionally, these biosignals are carried to the cell and affect the functioning of the cell [3]. In particular, exosomes can transmit biosignaling by receptor–ligand interactions, can be internalized by phagocytosis, and can fuse with the recipient cell’s membrane to deliver their content into the cytosol when exosomes attach to cells [4]. As a result, there are a variety of membrane proteins on exosomes, such as the intraluminal protein TSG-101 and the transmembrane proteins CD9, CD81, and CD63 [5]. While the crucial functions of exosomes have begun to be unraveled, there is increasing evidence that exosomes play an important role in physiology and pathophysiology. In physiology, exosomes are an important component of morphogen gradients. The key morphogen molecules Wingless (Wnt) and Hedgehog are carried by exosomes [6,7,8]. In pathophysiology, exosomes are not only involved in the development of cancer, but are also considered to be a kind of novel cancer marker. Al-Nedawi et al. found that levels of vascular endothelial growth factor (VEGF) improve after the release of glioblastoma-derived exosomes [9]. As a result, how to analyze the quantitation of exosomes is an issue for current biomedical knowledge and future medical diagnosis [10].

Nanoparticle tracking analysis (NTA) and flow cytometry techniques are typically used to quantify exosomes. NTA is a nanoparticle characterization technique that can visualize and measure suspended particles in a range of 10 to 1000 nm based on Brownian motion analysis. When a sample solution is placed on the sample table, a special laser beam illuminates the particles. The particles disperse the laser beam that is received by a microscope coupled with a digital camera. Thus, not only the trajectories of the particles are followed, but the hydrodynamic diameters of the particles are calculated [11]. However, larger protein aggregates in suspension might interfere with the accuracy of measurement and the concentration of particles needs to be diluted to a moderate range [12]. Flow cytometry is a technology that simultaneously measures and analyzes multiple physical characteristics of particles by scattered light [13]. Apart from the fact that flow cytometry accurately quantifies exosomes [14], the labeling of magnetic beads restricts their application. While the detection accuracy and sensitivity of NTA and flow cytometry are not low, there is a complicated operational process.

Label-free techniques, such as surface plasmon resonance (SPR) [12], nanoplasmonic exosome (nPLEX) assay [15], surface-enhanced Raman spectroscopy (SERS) [16], frequency locking optical whispering evanescent resonator (FLOWER), and depth scanning correlation are employed to quantify exosomes without using fluorescent labels [17,18]. SPR is considered a surface sensitive method used to detect different biomolecules such as DNA, RNA, proteins, and peptides [19,20,21,22]. A recent method based on self-assembly gold nanoislands (SAM-AuNIs) localized SPR was used to distinguish exosomes from microvesicles (MVs) [23]. Other groups have determined the concentration of exosomes in solution by SPR [12]. However, the sensitivity of the SPR biosensor is lower than that of the labeled biosensor.

The technique based on total internal reflected imaging ellipsometry (TIRIE) is a combination of conventional SPR and the imaging ellipsometry method. TIRIE was used as an analysis tool in real time for biomolecular interaction processes [24]. TIRIE is sensitive to the change in the interfacial refractive index of a biomolecule layer on the sensing surface [25,26]. Theoretically, TIRIE, under the total internal reflection mode, is 10 times more sensitive than conventional SPR [27,28]. TIRIE has been applied in numerous areas including early diagnosis of tumors, as a clinical therapy marker [29], interaction affinity analysis, and environmental pollution monitoring [24,30]. In the field of interaction affinity analysis, the biomolecule that is to be detected is called a target and the biomolecule that is used to immobilize the target is referred to as a ligand. There are two problems when detecting exosomes by TIRIE. On the one hand, although TIRIE has a mature detection process in proteins, an appropriate exosome detection system is lacking. On the other hand, exosomes do not have a single target site (protein), such as CD9 and CD63. Therefore, designing an exosome detection surface based on TIRIE and optimizing an appropriate target site on exosomes are issues.

**Scheme 1:**
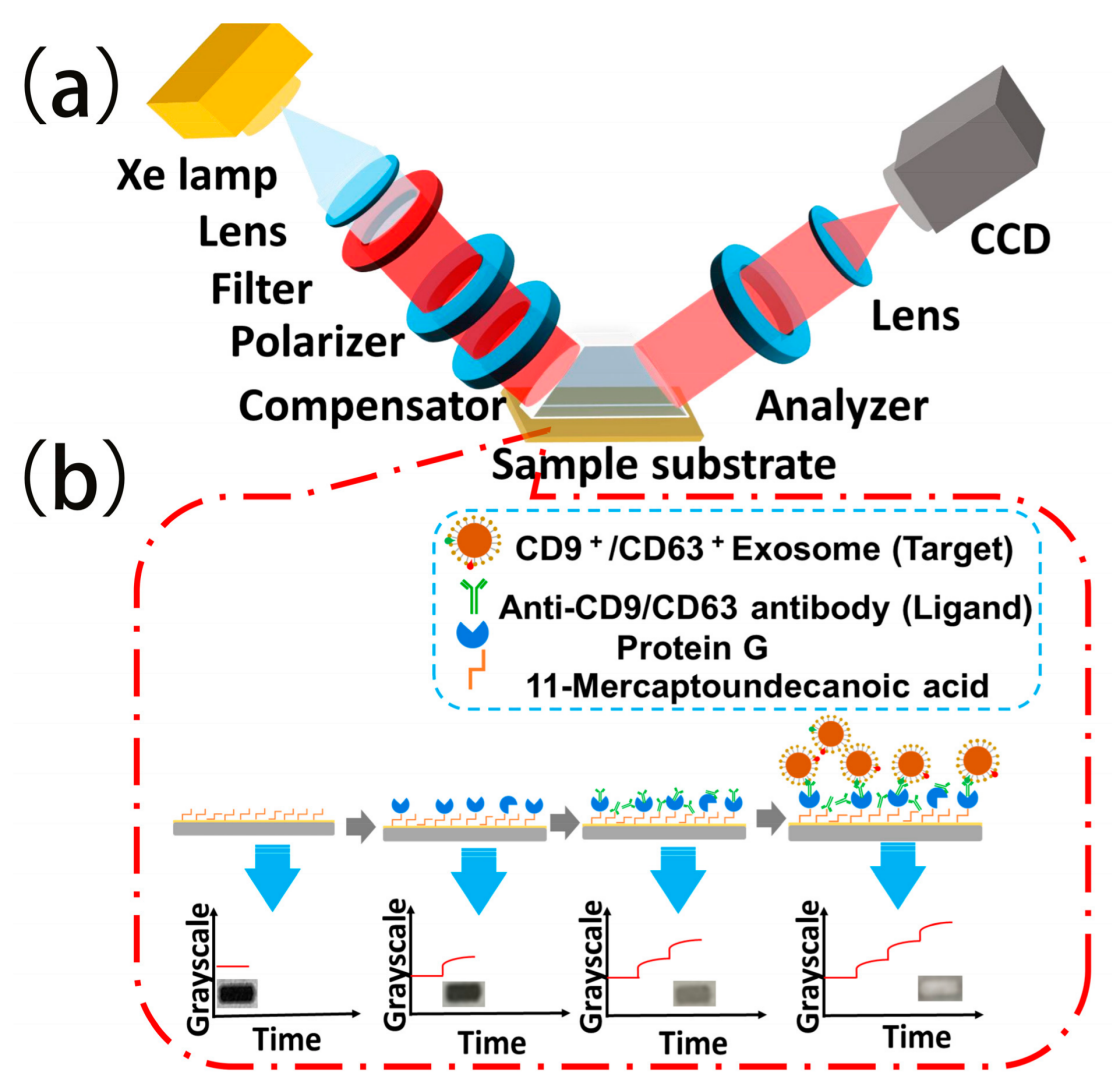
TIRIE for the detection of exosomes: (**a**) Schematic of TIRIE with the typical conventional polarizer–compensator–sample–analyzer configuration; (**b**) schematic of the bioreaction process on the sensing surface and the sensing signals recorded by TIRIE, correspondingly. The grayscale image shows the intensity of the light which is characteristic of the surface mass density distribution of the biomolecular layers on the surface. When the ligand in the solution interacts with the target, a complex is assembled on the surface by affinity and the surface mass density is changed. With the visualization by TIRIE, the changes on the surface are be transformed into grayscale and the targets in the solution can be verified quantitively.

In this work, we not only designed a sensing surface to detect exosomes, but also compared CD9 and CD63 on exosomes which are preferred as target sites of exosomes. As shown in Scheme 1, the sensing surface is modified with protein G and antibodies, which capture exosomes by exosome-specific membrane proteins CD63 and CD9. In the kinetic analysis, we introduced pseudo-first-order interaction kinetics to analyze the interaction between proteins and antibodies [31]. As far as we know, this is the first time that exosomes have been detected using TIRIE.

## 2. Materials and Methods

### 2.1. TIRIE

TIRIE is composed of a microfluidic microarray bioreactor and an imaging ellipsometry system operated under the total internal reflection mode. As is shown in Scheme 1a, a light beam with the wavelength of 633 nm from a Xe lamp and a wavelength selector goes through a polarizer and a quarter wave plate (compensator) successively, and is reflected from the substrate after penetrating a coupling prism perpendicularly. An evanescent wave that is exponentially decayed emerges on the gold ambient interface when the incident angle is larger than the total internal reflection angle. Mass density changes in biomolecules on the surface within this substrate modulate the elliptic parameters (psi and delta). The reflected light beam passes through another dichroic polarizer (analyzer) and is collected into the charge coupled device (CCD) camera in the format of a 16-bit grayscale image. If there is a biomolecule reaction occurring on the surface, the whole process of biomolecular interaction is visualized by the CCD in real time.

The structure of the microfluidic bioreactor has been described in the reference [31]. The microfluidic microarray bioreactor is composed of a microarray device and a peristaltic pump. The microarray has 24 independent interaction cells. Each cell is attached to microtubes via an inlet and an outlet port. Inlet ports are attached to the sample solutions, and outlet ports connect to a peristaltic pump. When a substrate surface adheres to the microarray, 24 independent interaction cells are formed. By the power of a peristaltic pump, biomolecules in the solution are pumped successively onto the surface.

### 2.2. Chemicals and Biological Samples

Anti-CD9 antibody [MEM-61] and anti-CD63 antibody [MEM-259] were purchased from Abcam. Recombinant protein G from Escherichia coli, ≥90% (protein G), anti-human IgG antibody (anti-IgG), 1-(3-Dimethylaminopropyl)-3-ethylcarbodiimidehydrochloride (EDC), N-hydroxyl-succinimide (NHS), and 11-mercaptoundecanoic acid (MUA) were all purchased from Sigma-Aldrich. ExoStd^TM^ lyophilized exosome standard (human serum) (Exo) was bought from Biovision (Milpitas, USA). Deionized water was obtained from a Milli-Q purification system (18.2 mΩ at 25 °C). Phosphate-buffered saline (PBS, pH 7.4) and PBST (containing 0.05% Tween-20) were prepared in deionized water.

### 2.3. Functionalization of Sensing Surface

The glass substrate coated with a gold film (50 nm) was cleaned in piranha solution (H_2_SO_4_:H_2_O_2_ = 3:1, *v*/*v*) for 30 min and then washed with deionized water and pure ethanol alternately five times. After being dried by pure nitrogen and cleaned with UV/Ozone ProCleaner^TM^Plus (BioForce Nanosciences, Ames, IA, USA) for 30 min, the glass substrate was immersed in an MUA ethanolic solution (10 mM) overnight. Finally, the modification glass substrate was rinsed by pure ethanol and deionized water alternately and was stored in pure ethanol. The surface modification procedure was carried out in a microfluidic microarray bioreactor, which was referred to in our previous works [24]. To stimulate the sensing surface bioactivity, a mixture solution prepared with NHS (0.05 mol/mL) and EDC (0.2 mol/mL) was passed across the sensing surface at 1 μL/min for 8 min, followed by washing with PBST.

### 2.4. The Surface Processing of Biomolecules

The protein G was immobilized on the functionalized sensing surface to modify the ligand. Firstly, the protein G flowed at 1 μL min/mL for 8 min, followed by washing with PBST. Then, the ligand was injected on the surface at 1 μL min/mL for 20 min and then rinsed with PBST for 20 min. Finally, exosomes were delivered at 1 μL/min for 30 min, followed by washing with PBST for 10 min.

### 2.5. Dynamic Light Scattering Measurements

The diameter of exosomes was characterized by dynamic light scattering (Zetasizer Nano, Malvern Panalytical Ltd, Malvern, UK) which measured Brownian motion to determine the size of the particles. By illuminating the particles with the laser, the fluctuations in the intensity of the diffused light were analyzed.

## 3. Results and Discussion

### 3.1. Characterization of the Diameter and Surface Protein of Exosomes

**Figure 1:**
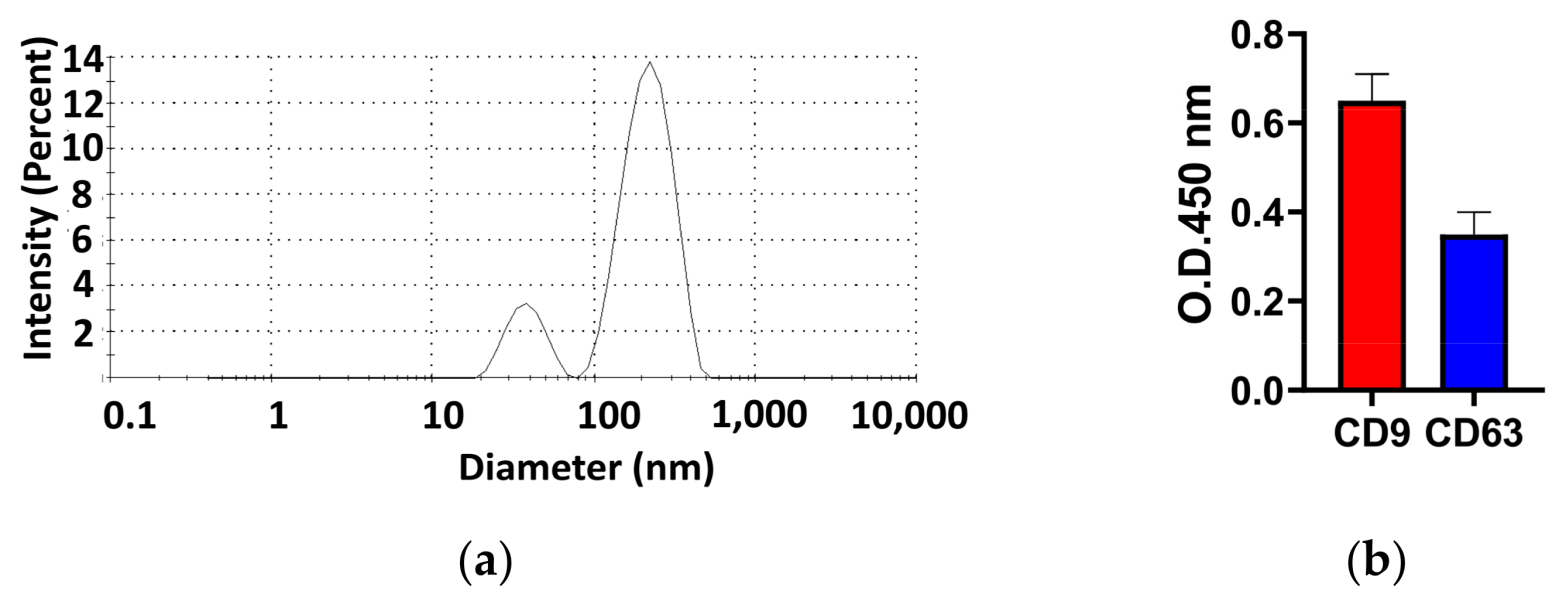
The characterization of the diameter and the surface proteins of exosomes. (**a**) The diameter of exosomes was characterized by a Zetasizer Nano. (**b**) The level of CD9 or CD63 surface protein on exosomes was characterized by ELISA.

The diameter of exosomes was characterized by a Zetasizer Nano and CD9 and CD63 surface proteins were quantified by ELISA. As shown in Figure 1a, peak intensity accumulated in two (220 nm ± 70 nm and 38 nm ± 10 nm). The proportion of the area under the curve of the higher peak intensity was 84%. However, the proportion of the area under the curve of the lower peak intensity was 16%. Furthermore, the distribution range of diameters in the higher peak intensity was between 150 and 290 nm and that in the lower peak intensity was between 50 and 150 nm. In further experiments, the surface proteins (CD9 and CD63) on exosomes were characterized by an ELISA assay, as shown in Figure 1b. The optical density (OD) value of CD9 (0.7 ± 0.08) was 1.8 times greater than CD63 (0.38 ± 0.07), which suggested that the number of CD9 proteins on exosomes exceeded the number of CD63 proteins on exosomes derived from human serum.

### 3.2. Design of Sensing Surface

#### 3.2.1. Immobilization of Antibody by Protein G

**Figure 2:**
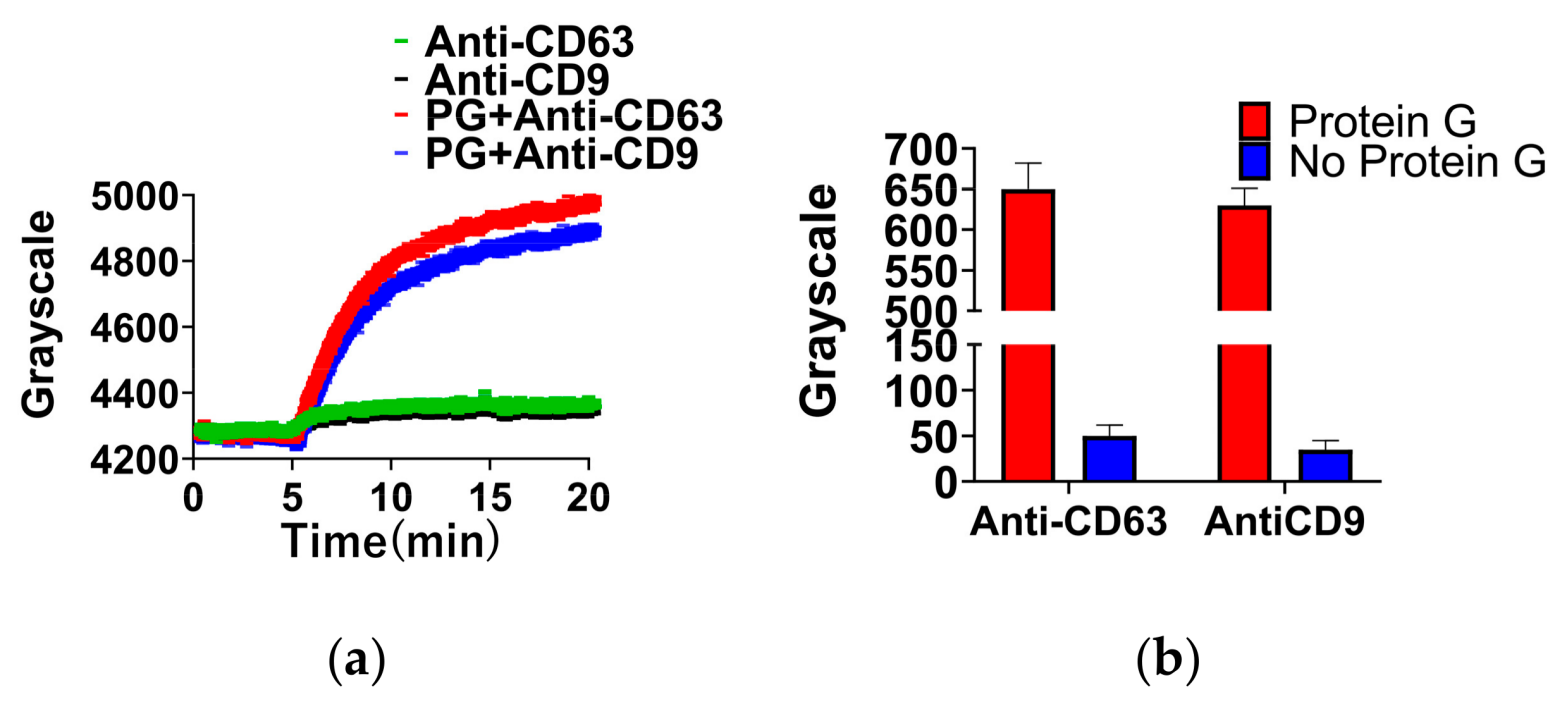
Detection of antibodies with or without protein G by TIRIE. (**a**) The real-time curve of the sensing surface in four cases. (**b**) The sensing signal variation of antibodies on the surface with or without protein G.

Protein G was utilized to improve ligand quantity in order to enhance the ligand detection signal. As shown in Figure 2a, the sensing signal of anti-CD63 or anti-CD9 with protein G was significantly improved. As shown in Figure 2b, comparing the red and blue bars, the increment in anti-CD63 (650 ± 40) or anti-CD9 (640 ± 20) with protein G is at least twelve times that of anti-CD63 (50 ± 10) or anti-CD9 (40 ± 10) without protein G. These results indicated that protein G enhanced the amount of anti-CD9 or anti-CD63 adsorption. On the one hand, protein G is a bacterial cell wall protein isolated from group G *Streptococci* [32]. DNA sequencing of native protein G identified two IgG-binding domains and sites for albumin [32]. On the other hand, anti-CD9 and anti-CD63 have two IgG-binding domains because these proteins are the isotype of IgG1. Therefore, protein G has the ability to bind anti-CD9 or anti-CD63 selectively.

#### 3.2.2. Optimum Concentration of Antibody

**Figure 3:**
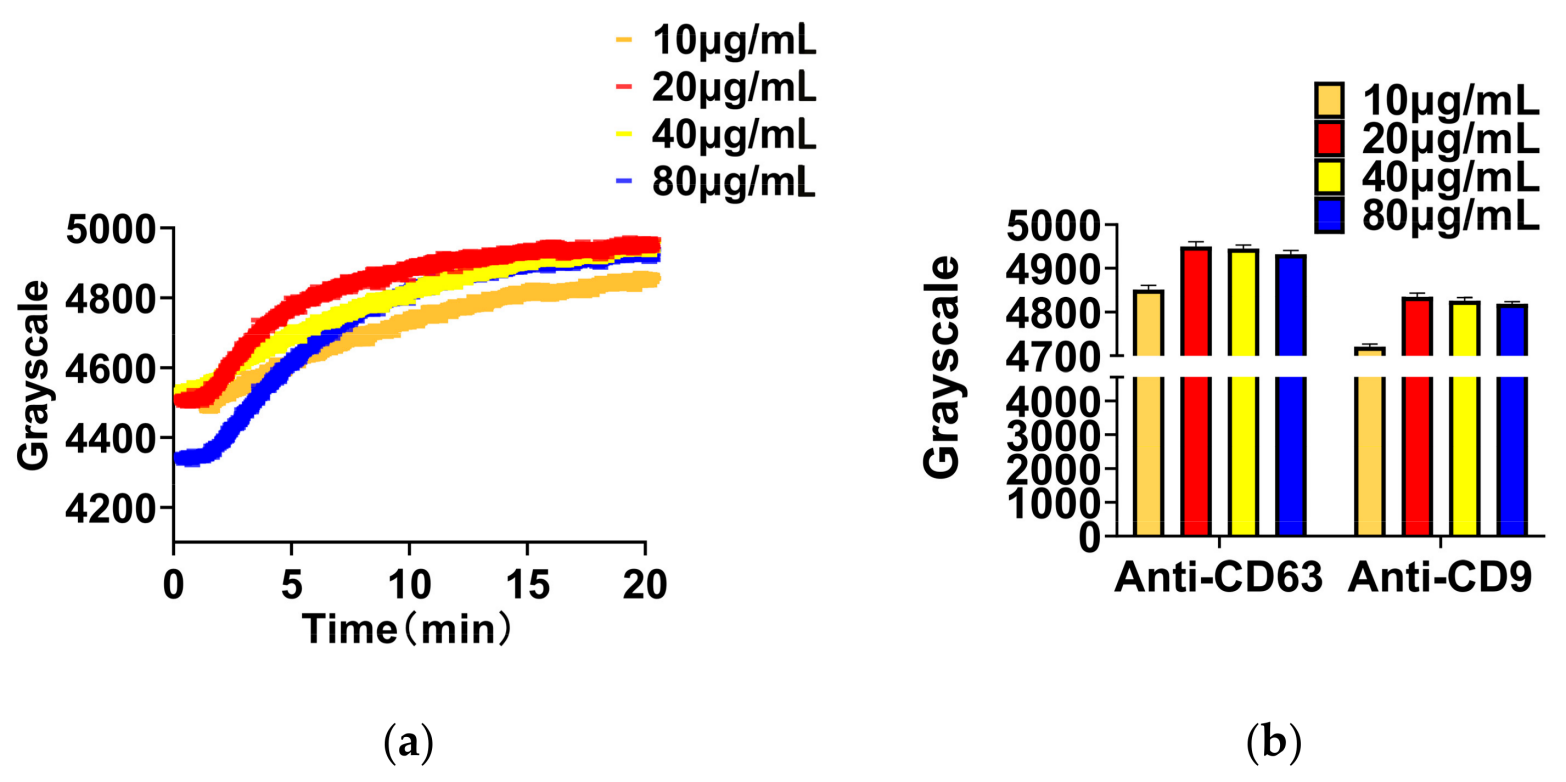
Detection of different concentrations of antibody by TIRIE. (**a**) Four real-time curves for the interaction between protein G and anti-CD9/anti-CD63 in four concentrations, respectively. (**b**) The sensing signal of four different concentrations of anti-CD9 and CD63.

The amount of absorption of exosomes depends on the number of antibodies on the sensing surface. Therefore, a proper concentration (saturated concentration) of antibody is necessary. The concentration of antibodies was diluted in four levels, 10 μg/mL, 20 μg/mL, 40 μg/mL, and 80 μg/mL by PBST. The real-time curve of antibody adhesion is shown in Figure 3a. As shown in Figure 3b, the concentration of 20 μg/mL produced the highest sensing signal for both anti-CD9 and anti-CD63. However, the detection signal did not increase as the concentration increased. One the one hand, too low a concentration of antibody (10 μg/mL) may bring exosomes into direct contact with the surface because of the large number of virgin sites on the surface. On the other hand, too high a concentration of antibody (80 μg/mL) may reduce the number of exosomes on the surface due to the appearance of steric hindrance [33]. Therefore, the concentration of 20 μg/mL of anti-CD9 or anti-CD63 is not only the saturated adsorption concentration on the sensing surface, but was also employed as the normal concentration in the follow-up experiment.

### 3.3. Detection and Analysis of Exosomes

**Figure 4:**
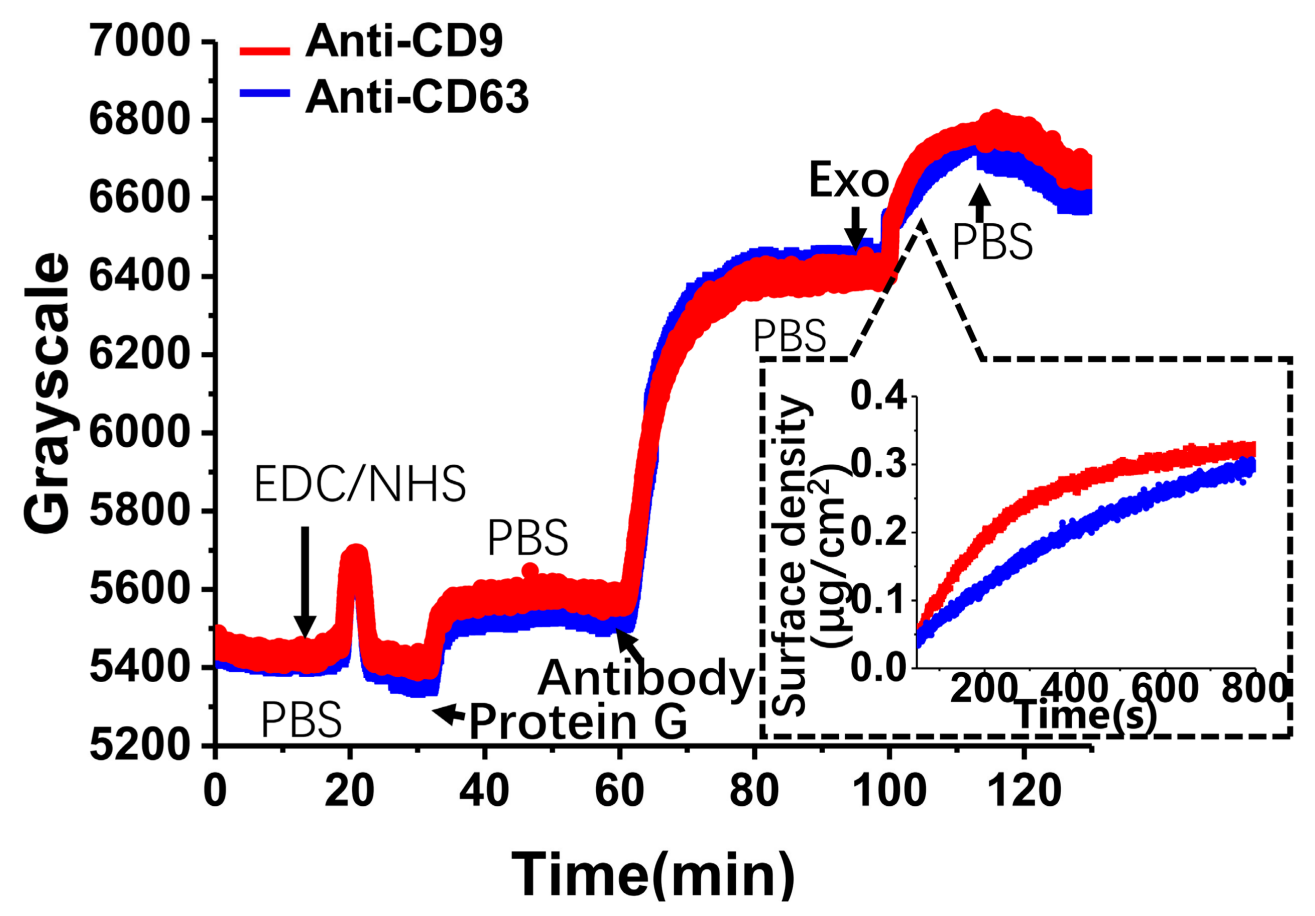
The real-time curve for the whole biological process on the sensing surface. The inset in the figure clearly illustrates the specific binding process between two types of antibodies (anti-CD9 and anti-CD63) and exosomes.

The whole real-time procedure of exosome adhesion was recorded by TIRIE. As shown in Figure 4, EDC/NHS was pumped into the flow cell in the first 20 min. After washing with PBS, protein G was immobilized on the activated surface at a concentration of 100 μg/mL for a further 30 min. Antibodies (anti-CD9 or anti-CD63) were injected on the sensing surface during the next 20 min. After washing with PBS, exosomes at a concentration of 200 μg/mL were delivered to the surface within 15 min. For protein G, the grayscale increased by 200. Antibodies showed an increase of 900 grayscale. Finally, for exosomes, the grayscale increased by 300. In fact, TIRIE responded approximately linearly to the variation in surface mass density (μg/cm^2^) of the protein layer [34]. The relationship between the TIRIE signal, δI, and the surface mass density of the protein layer, δΓ, can be given by

(1) δI∝δΓ=δm·M

where δm and *M* are the surface amount density of the protein layer and the protein mass, respectively. Here, we suppose that the exosome mass is approximated to the protein mass. Previous studies reported the mass of all proteins in exosomes. Thus, according to Equation (1) the TIRIE signal (grayscale) of exosomes can be transformed into surface mass density (μg/cm^2^). As shown in the inset of Figure 4, exosomes captured by anti-CD9 have a higher surface density than exosomes captured by anti-CD63 after 15 min.

The surface density, the average surface occupied, and the average distance between exosomes were estimated. As shown in the Figure 4 inset, the surface mass density is 0.3 μg/mL, shown by the blue curve, up to 800 s. Assuming the diameter of exosomes is 200 nm, the density of exosomes is 7.2 × 10^7^ (particles/cm^2^). The surface size of attached exosomes is 3 mm ± 0.1 mm × 0.5 mm ± 0.1 mm. Therefore, the number of exosomes is 1.1 × 10^5^. Supposing the surface is separated into a set number of squares whose side length is 200 nm, the number of squares is 3.7 × 10^7^. As a result, the number of squares is 34 times the number of exosomes. Then, supposing all exosomes are evenly distributed on the surface, the average distance between bound exosomes is 44 nm ± 0.5 nm.

**Figure 5:**
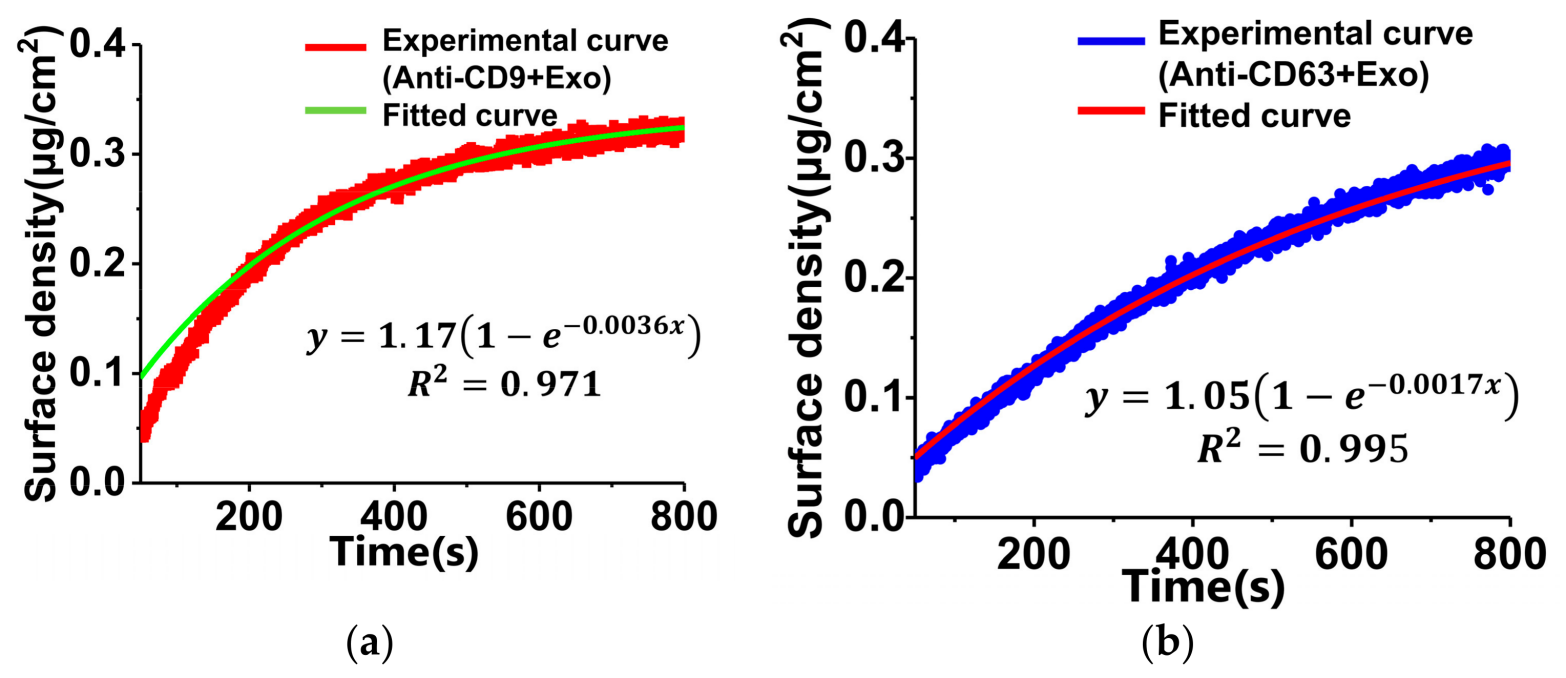
The experimental and fitted curve for the interaction between antibodies and exosomes. (**a**) The red curve represents the interaction between exosomes and anti-CD9; (**b**) the blue curve represents the interaction between exosomes and anti-CD63.

We analyzed the $K_{D}$ of the ligand–analyte interaction on the sensing surface to compare the detection ability of TIRIE with anti-CD9 with exosomes and anti-CD63 with exosomes. There are two hypotheses. First, the binding ratio of antibodies and exosomes is 1 to 1. Second, the interaction between antibodies and exosomes represents pseudo-first-order interaction kinetics. Therefore, the fitting model can be expressed by

(2) $$\delta \;y=A_{1}/A_{2}\left(1-e^{-\mathrm{kx}}\right)$$

where A_1_/A_2_ is the slope of the fitting curve. For TIRIE, a typical interaction between the ligand and the analyte on the surface follows: ligand + analyte ⇌ ligand − analyte [35]. Considering the pseudo-first-order interaction, the surface density can be expressed by [34]

(3) $$\Gamma_{analyte}=\frac{\Gamma_{analyte}{}_{0}\cdot c_{analyte}}{K_{D}+c_{analyte}}$$

where $\Gamma_{analyte}{}_{0}$ is the initial surface density of the ligand before the interaction, $K_{D}$ is the dissociation equilibrium constant of the interaction, and $c_{analyte}$ is the concentration of the analyte in solution. As shown in Figure 5, the square numbers (R^2^) of two curves are greater than 0.97, suggesting that an appropriate degree of the pseudo-first-order interaction model curves is consistent with the experimental curves. In terms of Equation (2), the yellow curve in Figure 5a has a larger A_1_/A_2_ than the red curve in Figure 5b. According to Equations (1) and (3), a higher Γ leads to a lower $K_{D}$, and a smaller $K_{D}$ suggests a stronger binding affinity between ligand and analyte [34]. As shown in Figure 5, the red curve (anti-CD9) has a greater surface density than the blue curve (anti-CD63). Therefore, the interaction between anti-CD9 and exosomes has a stronger binding affinity than the interaction between anti-CD63 and exosomes.

**Figure 6:**
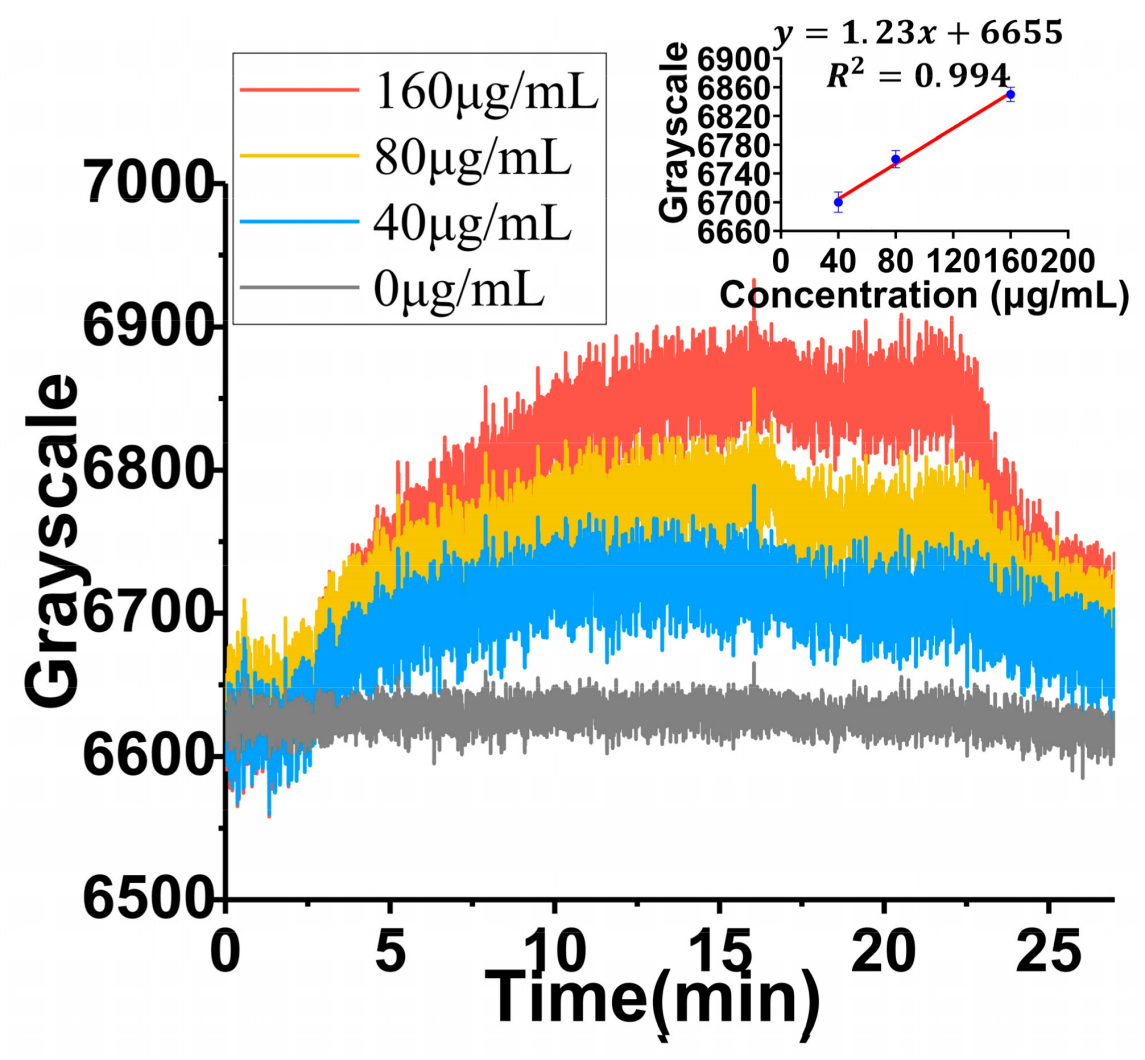
Detection of different concentrations of exosomes by TIRIE based on anti-CD9. The inset is the calibration curves of exosomes in three concentrations (40 μg/mL, 80 μg/mL, and 160 μg/mL).

In order to verify the detection range (concentration) of exosomes in TIRIE, three different concentrations of exosomes (40 μg/mL, 80 μg/mL, 160 μg/mL) were considered as targets. Since the interaction between the anti-CD9 and exosomes showed a higher affinity for binding, anti-CD9 was employed as a ligand in this part. As shown in Figure 6, as the exosome concentration increases, the TIRIE signal increases steadily. The TIRIE signal changes from 100 grayscale to 250 grayscale. According to the calibration curve from the Figure 6 inset, the LOD of TIRIE is 0.41 μg/mL assuming a 95% confidence level. The calculation of LOD is based on the calibration curve with S/σ = 3.3, where S and σ are the slope of the calibration curves of exosomes (Figure 6 inset) and the standard deviation of 20 independent blank control measurements, respectively.

One probable reason why the interaction between exosomes and anti-CD9 has a $K_{D}$ greater than that between exosomes and anti-CD63 is that the CD9 protein expressed itself more on the exosome surface. This reason is demonstrated in Figure 1b, where the expression of the CD9 protein exceeds the CD63 protein on exosome surfaces from human serum. The more proteins that express themselves on exosomes, the more binding sites are linked to the antibody specifically. Additionally, more binding sites indicate a higher binding probability. In addition, proteomic comparison results of exosomes from 2016 indicated that the amount of CD9 is more than CD63 at a moderate (10,000 g) centrifugation speed [36].

## 4. Conclusions

In summary, we not only designed a sensing surface to capture exosomes successfully based on TIRIE, but also compared the $K_{D}$ of the interaction between the antibody and CD9 and CD63 surface proteins on exosomes by introducing the pseudo-first-order interaction kinetics mode. Firstly, the antibody detection signal (anti-CD9 or anti-CD63) with protein G is 1.14 times higher than that without protein G. Secondly, the distance between exosomes adhered on surfaces is 44 nm ± 0.5 nm when the concentration of exosomes is 200 μg/mL. In addition, the interaction between the CD9 surface protein on exosomes and anti-CD9 has a smaller $K_{D}$ than the interaction between the CD63 surface protein on exosomes and anti-CD63. We speculate that a higher expression level of CD9 on exosomes is the reason why the interaction of exosomes and anti-CD9 has a smaller $K_{D}$. Therefore, CD9 is more suitable than CD63 as a surface biomarker of exosomes. Finally, the LOD of TIRIE to detect exosomes is 0.41 μg/mL. This indicates that TIRIE is suitable as a tool to study the interaction process between proteins and exosomes.

## Data Availability

Not applicable.
